# Plasma levels of tumour necrosis factor and its soluble receptors correlate with clinical features and outcome of Hodgkin's disease patients.

**DOI:** 10.1038/bjc.1998.391

**Published:** 1998-06

**Authors:** K. Warzocha, J. Bienvenu, P. Ribeiro, I. Moullet, C. Dumontet, E. M. Neidhardt-Berard, B. Coiffier, G. Salles

**Affiliations:** Service d'HÃ©matologie, Centre Hospitalier Lyon-Sud, Hospices Civils de Lyon and UPRES-JE 1879 HÃ©mopathies, LymphoÃ¯des malignes, UniversitÃ© Claude Bernard, Pierre-BÃ©nite, France.

## Abstract

A prospective study was performed to assess the use of plasma measurement of tumour necrosis factor (TNF), lymphotoxin alpha (LT alpha) and their soluble receptors (p55 and p75) for prognostic risk assignment in 61 patients with Hodgkin's disease. Plasma levels of TNF, p55 and p75, but not of LT alpha, were higher in Hodgkin's disease patients than in healthy controls. Plasma levels of TNF, p55 and p75 were associated with several prognostic factors for Hodgkin's disease, including those related to the host (age, performance status) and to the tumour (disease stage, extranodal site involvement, bulky tumour, serum levels of LDH and beta2-microglobulin, histology). Elevated plasma levels of TNF, p55 and p75 were also associated with several parameters reflecting an immune activation, including the presence of B symptoms, elevated serum levels of gammaglobulins, alkaline phosphatase and fibrinogen, as well as peripheral monocytosis, anaemia and low serum albumin levels. Finally, elevated TNF ligand receptor plasma markers were associated with a lower incidence of complete response to therapy and predicted shorter free-from-progression survival and overall survival of the patients. These results indicate that the plasma levels of TNF and its soluble receptors correlate with clinical features and outcome of patients with Hodgkin's disease.


British Journal of Cancer (1998) 77(12), 2357–2362
© 1998 Cancer Research Campaign

### Plasma levels of tumour necrosis factor and its soluble receptors correlate with clinical features and outcome of Hodgkin's disease patients

**K Warzocha[1], J Bienvenu[2], P Ribeiro[1], I Moullet[1], C Dumontet[1], EM Neidhardt-Berard[1], B Coiffier[1] and G Salles[1]**

[1]Service d'Hématologie and [2]Laboratoire d'Immunologie, Centre Hospitalier Lyon-Sud, Hospices Civils de Lyon and UPRES-JE 1879 'Hémopathies Lymphoïdes malignes', Université Claude Bernard, 69495 Pierre-Bénite, France

**Summary** A prospective study was performed to assess the use of plasma measurement of tumour necrosis factor (TNF), lymphotoxin alpha (LTα) and their soluble receptors (p55 and p75) for prognostic risk assignment in 61 patients with Hodgkin's disease. Plasma levels of TNF, p55 and p75, but not of LTα, were higher in Hodgkin's disease patients than in healthy controls. Plasma levels of TNF, p55 and p75 were associated with several prognostic factors for Hodgkin's disease, including those related to the host (age, performance status) and to the tumour (disease stage, extranodal site involvement, bulky tumour, serum levels of LDH and β2-microglobulin, histology). Elevated plasma levels of TNF, p55 and p75 were also associated with several parameters reflecting an immune activation, including the presence of B symptoms, elevated serum levels of gammaglobulins, alkaline phosphatase and fibrinogen, as well as peripheral monocytosis, anaemia and low serum albumin levels. Finally, elevated TNF ligand receptor plasma markers were associated with a lower incidence of complete response to therapy and predicted shorter free-from-progression survival and overall survival of the patients. These results indicate that the plasma levels of TNF and its soluble receptors correlate with clinical features and outcome of patients with Hodgkin's disease.

**Keywords:** tumour recrosis factor; ligand; receptor; Hodgkin's disease; prognosis

An important issue in Hodgkin's disease (HD) is the identification of prognostic factors that influence response to therapy and outcome of the patients. The most common factors that have been found to be associated with an unfavourable outcome include advanced stage, presence of extranodal disease and bulky tumour, mixed cellularity or lymphocyte depletion histology, age above 45 years, presence of B symptoms, male gender, elevated serum levels of LDH, β2-microglobulin, alkaline phosphatase and low serum albumin levels. However, despite the presence of several prognostic indices for HD, there is no agreement on the definition of sufficiently high-risk groups of patients for whom the prognosis is especially poor and for whom up-front high-dose therapy should be advised (Wagstaff et al, 1988; Straus et al, 1990; Proctor et al, 1991; Hasenclever et al, 1995; Lee et al, 1997). Thus, the search for more discriminating prognostic factors identifying vulnerable patients with a high risk of resistance or relapse is still an open challenge.

Hodgkin's disease is frequently accompanied by systemic symptoms, increased erythrocyte sedimentation rate, hyperfibrinogenaemia, thrombocytosis, anaemia and eosinophilia. Histologically, HD is characterized by the presence of a low proportion of Hodgkin's and Reed–Sternberg (H-RS) cells, with a predominant reactive lymphocyte population and mixed degrees of fibrosis or sclerosis within surrounding lymphoid tissue. Populations of H-RS cells and reactive cells are both capable of producing a panel of different cytokines and receptors (Gruss and Dower, 1995). It seems probable therefore that the major clinical and histopathological features of HD reflect the production of cytokines by either the neoplastic or the reactive cell populations.

Tumour necrosis factor (TNF) was originally identified as cachectin, a soluble factor involved in weight loss, fever and anaemia, but it was soon demonstrated that TNF is a central mediator of inflammatory processes, generating a cytokine cascade that includes the production of interleukin 1, interleukin 6 and other secreted proteins, as well as TNF itself (Warzocha et al, 1995; Bazzoni and Beutler, 1996). In addition, TNF and LTα (formerly known as TNF-β) have been recently identified to participate in the development and in the function of normal lymphoid tissues (Le Hir et al, 1996; Pasparakis et al, 1996). Two receptors, p55 (TNF-RI) and p75 (TNFR-II), mediate the effects of TNF and LTα on target cells through several pathways leading to cell activation, proliferation or apoptotic death. Soluble forms of both receptors have been identified in biological fluids and modulate the effects of the cytokines (Warzocha et al, 1995; Bazzoni and Beutler, 1996). The observation of structural similarities between several molecules and TNF or its receptors has resulted in the definition of a larger family of molecules, including TNF, LTα, LTβ, Fas and the antigens CD40, CD30 and CD27, many of these molecules being involved in the immune system regulation.

Stimulation of the CD30 antigen, a member of the TNF receptor family, regarded as being a peculiar attribute of H-RS cells, resulted in inducible secretion of both TNF and LTα by these cells (Gruss et al, 1995). Furthermore, H-RS cells express several TNF receptors, including those specific for TNF and LTα (Gruss and Dower, 1995). In addition, it is of interest that both cytokines and the soluble forms of their receptors were found to be elevated in patients with non-Hodgkin's lymphoma (NHL) and that TNF, p55 and p75 plasma levels represented valuable prognostic markers in

*Received 18 June 1997*
*Revised 4 December 1997*
*Accepted 8 December 1997*

*Correspondence to:* G Salles, Service d'Hématologie, Centre Hospitalier Lyon-Sud, 69495 Pierre Benite, France those individuals (Salles et al, 1996; Warzocha et al, 1997). In the current prospective study, we demonstrated that elevated plasma levels of TNF and its soluble receptors were present in a subset of HD patients who had several adverse prognostic factors at the time of diagnosis. In addition, elevated levels strongly predicted shorter free-from-progression (FFP) survival and overall survival of the patients. These data suggest the possible use of TNF and its soluble receptors as serum markers for risk assignment in HD.

### METHODS

### Patients

From October 1991 to November 1996, 61 recently diagnosed HD patients (23 women and 38 men) were consecutively admitted to our department and enrolled in this study. The median patient age was 37 years (range 15–75). Patients with active bacterial or fungal infection and those who tested positive for the human immunodeficiency virus were excluded from the study. Patients with a previous history of autoimmune disease as well as patients who had received steroid therapy were also excluded from the analysis. In all patients, the diagnosis was based on lymph node histology, with the lymphocyte predominance subtype being found in three patients, nodular sclerosis in 38, mixed cellularity in 16 and lymphocyte depletion in four patients. The initial medical evaluation consisted of complete history and physical examination, radiographic examination of the chest, computerized tomographic scan of the chest, abdomen and pelvis, and blood chemistry. The extent of disease and the presence of B symptoms were categorized according to the Ann Arbor staging classification. Bulky disease was defined by a mass greater than 10 cm in its largest dimension or by a mediastinal mass greater than one-third of the maximal thoracic diameter on a standing chest radiograph. Clinical characteristics of the patients included in the present study are shown in Table 1.

### Treatment

Treatment was defined according to the initial HD stage. Among these 61 patients, five underwent radiotherapy alone, 41 were treated with chemotherapy and radiotherapy, and 15 patients received only chemotherapy. Chemotherapy regimens consisting of MOPP–ABVD alternated, MOPP/ABV-hybrid and ABVD-like protocols were used, respectively, in 17, 9 and 30 patients. A complete response to treatment was defined as the disappearance of all clinical manifestations of the disease and normalization of all laboratory values. Free-from-progression survival was calculated from the date of treatment initiation until relapse, disease progression or the last follow-up evaluation. Overall survival was measured as the time between the beginning of treatment and death or the date of the last follow-up evaluation.

### Evaluation of plasma cytokine and receptor levels

All samples were collected before treatment initiation using sterile tubes containing EDTA to prevent further release of cytokines before analysis. Plasma samples were tested using ELISA kits for TNF (Medgenix Diagnostics, Fleurus, Belgium), LTα (R&D Systems, Minneapolis, MN, USA), p55 and p75 (Roche, Basle, Switzerland). The detection limits of the ELISA tests used were 3 pg $ml^{-1}$ for TNF, 7 pg $ml^{-1}$ for LTα, 0.1 ng $ml^{-1}$ for p55 and 1 ng $ml^{-1}$ for p75. Plasma samples from 20 healthy subjects, including five women and 15 men, with a median age of 30 years (range 22–50), served as controls for the detection of these proteins.

**Table 1** Characteristics of patients with Hodgkin's disease and association of the TNF, p55 and p75 plasma levels (mean values) with clinical features of the disease

| Characteristics | Number of patients | TNF | p55 | p75 |
|---|---|---|---|---|
| Age | | **<0.02** | **<0.0001** | **<0.005** |
| 60 years | 55 | 32.2 | 2.9 | 5.1 |
| > 60 years | 6 | 66.3 | 6.0 | 12.3 |
| Performance status (ECOG) | | **<0.0005** | **<0.0001** | **<0.0005** |
| < 2 | 53 | 30.1 | 2.8 | 4.9 |
| 2 | 8 | 71.5 | 5.4 | 12.2 |
| Weight loss | | **<0.0001** | **<0.0001** | **<0.0001** |
| Absent | 50 | 28.3 | 2.8 | 4.4 |
| Present | 11 | 68.6 | 5.1 | 12.2 |
| Fever | | **<0.0001** | **<0.0005** | **<0.0005** |
| Absent | 39 | 23.1 | 2.5 | 3.9 |
| Present | 22 | 57.6 | 4.3 | 9.2 |
| Sweats | | **<0.01** | **<0.001** | **<0.005** |
| Absent | 47 | 29.9 | 2.8 | 4.7 |
| Present | 14 | 54.4 | 4.5 | 9.6 |
| Serum albumin | | **<0.0001** | **<0.0001** | **<0.0001** |
| ≥ 35 g $l^{-1}$ | 49 | 26.7 | 2.7 | 4.3 |
| < 35 g $l^{-1}$ | 12 | 71.8 | 5.3 | 12.0 |
| Haemoglobin | | **<0.001** | **<0.001** | **<0.005** |
| ≥ 12 g $dl^{-1}$ | 37 | 24.7 | 2.6 | 4.2 |
| < 12 g $dl^{-1}$ | 24 | 52.3 | 4.1 | 8.4 |
| Ann Arbor stage | | **<0.0001** | **<0.05** | **<0.005** |
| I, II | 39 | 23.9 | 2.8 | 4.2 |
| III, IV | 22 | 56.2 | 3.9 | 8.7 |
| Serum LDH | | **<0.05** | **NS** | **<0.01** |
| ≥ 1 × Normal | 48 | 31.1 | 3.0 | 4.8 |
| > 1 × Normal | 13 | 52.1 | 4.0 | 9.5 |
| Serum 2-microglobulin | | **<0.0001** | **<0.0001** | **<0.0001** |
| ≥ 3.0 mg $l^{-1}$ | 53 | 27.1 | 2.8 | 4.9 |
| > 3.0 mg $l^{-1}$ | 8 | 91.3 | 5.7 | 15.4 |
| Bulky tumour (10 cm) | | **<0.0005** | **<0.005** | **NS** |
| Absent | 54 | 30.5 | 2.9 | 5.6 |
| Present | 7 | 74.1 | 5.1 | 7.8 |
| Extranodal involvement | | | | |
| Bone marrow | | **<0.005** | **<0.0001** | **<0.0001** |
| Absent | 57 | 32.4 | 2.9 | 5.1 |
| Present | 4 | 80.0 | 6.9 | 16.7 |
| Spleen | | **<0.0001** | **<0.0005** | **<0.0001** |
| Absent | 51 | 27.8 | 2.8 | 4.7 |
| Present | 10 | 74.8 | 5.1 | 13.3 |
| Liver | | **<0.05** | **<0.05** | **<0.0005** |
| Absent | 55 | 33.1 | 3.0 | 5.0 |
| Present | 6 | 58.3 | 4.5 | 13.4 |
| Pleura | | **<0.05** | **<0.05** | **<0.005** |
| Absent | 56 | 32.8 | 3.0 | 5.2 |
| Present | 5 | 66.0 | 4.9 | 12.6 |
| Histology | | | | |
| Lymphocyte predominance | 3 | 13.3 | 2.5 | 3.2 |
| Nodular sclerosis | 38 | 28.3 | 2.9 | 4.4 |
| Mixed cellularity | 16 | 49.3 | 4.0 | 9.8 |
| Lymphocyte depletion | 4 | 66.0 | 3.1 | 6.1 |

Student's *t*-test *P*-value in bold; NS denotes non-significant.

© Cancer Research Campaign 1998

### Statistical analyses

Comparison of the cytokine and receptor plasma values between the patients and healthy controls, and all differences in means were tested using the paired Student's *t*-test. Linear correlations were determined using the Pearson test. Univariate analysis was performed using Yates corrected $\chi^2$ test. The FFP survival and overall survival were estimated using the Kaplan–Meier method, and statistical differences were assessed using the log-rank test. Statistical analysis was performed using the Statistica software (Statsoft, Tulsa, OK, USA).

### RESULTS

### Plasma cytokine and receptor levels

TNF, LTα, p55 and p75 were detectable in all patients' plasma samples collected at diagnosis before treatment. Their mean values were not statistically different in men compared with women. TNF mean plasma values were higher in HD patients (mean 35.5 pg $ml^{-1}$, range 9–142 pg $ml^{-1}$) than in healthy controls (mean 11 pg $ml^{-1}$, range 5–14 pg $ml^{-1}$) ($P < 0.0001$). LTα mean plasma values were 10.5 pg $ml^{-1}$ (range 7.0–18.8 pg $ml^{-1}$) in patients, and this was not statistically different from the healthy controls' values (mean 9.5 pg $ml^{-1}$, range 7.0–12.5 pg $ml^{-1}$). Soluble p55 receptor plasma levels obtained in the patients ranged from 1.2 ng $ml^{-1}$ to 11.5 ng $ml^{-1}$ (mean 3.2 ng $ml^{-1}$), whereas, in 20 healthy controls, values ranged from 1.5 ng $ml^{-1}$ to 3.4 ng $ml^{-1}$ (mean 2.3 ng $ml^{-1}$) ($P < 0.0001$). Soluble p75 receptor plasma levels ranged from 2.2 ng $ml^{-1}$ to 38.4 ng $ml^{-1}$ (mean 5.8 ng $ml^{-1}$), whereas, in controls, values ranged from 1.7 ng $ml^{-1}$ to 5.5 ng $ml^{-1}$ (mean of 4.0 ng $ml^{-1}$) ($P < 0.0001$).

### Plasma cytokine and receptor levels and other prognostic variables

A linear correlation was observed between the plasma concentrations of TNF, p55 and p75 ($P < 0.0001$ for each test), but not with LTα. LTα plasma levels were not associated with any major prognostic risk factor for HD and thus were not considered in the further analysis.

Elevated plasma levels of TNF, p55 and p75 were significantly associated with adverse prognostic factors at presentation, including those related to the host (age over 60 years, poor performance status), those related to the tumour (disease stage III/IV, presence of bulky tumour, extranodal disease involvement and increased serum levels of β2-microglobulin and LDH) and those related to the host–tumour relationship (presence of B symptoms, low haemoglobin and serum albumin levels) (Table 1). Elevated plasma levels of TNF were also associated with hyperfibrinogenaemia ($P < 0.005$), higher serum levels of gammaglobulins ($P < 0.05$) and increased peripheral monocytosis ($P < 0.001$), while elevated plasma levels of p75 were associated with increased serum levels of alkaline phosphatase ($P < 0.02$). Finally, TNF, p55 and p75 plasma levels were lower in nodular sclerosis than in the mixed cellularity histological subtype ($P < 0.02$, $P < 0.05$ and $P < 0.002$ respectively).

### Plasma cytokine and receptor levels and patient outcome

To search for a possible prognostic significance of the TNF, p55 and p75 plasma levels, values greater than those observed in healthy controls were established as cut-off points for discrimination of HD patients with elevated cytokine/receptor plasma levels (14 pg $ml^{-1}$, 3.4 ng $ml^{-1}$ and 5.5 ng $ml^{-1}$ respectively). With this criteria, we found elevated plasma levels of TNF in 48 (78%), of p55 in 22 (35%) and of p75 in 20 (32%) HD patients.

Among these 61 patients, 55 (90%) achieved a complete response to treatment. Out of 61 patients, 12 (19%) patients have experienced disease progression, while 49 (81%) have not, and seven (11%) patients have died. The median follow-up of the patients remaining alive is 27 months. A complete response to therapy was of a high predictive value for both FFP survival and overall survival of HD patients included in the present analysis (log-rank test $P < 0.0001$). Elevated plasma levels of TNF, p55 and p75 were markedly associated with a lower incidence of complete response to therapy ($P < 0.01$, $P < 0.0005$ and $P < 0.0001$ respectively). Both p55 and p75 plasma levels, but not TNF plasma levels, were also associated with shorter FFP survival and shorter overall survival in univariate analysis (log-rank test $P < 0.05$). Among the established prognostic factors for HD, only age over 60 years (but not over 45 years), advanced disease stage, poor performance status, low serum albumin levels and extranodal disease involvement (bone marrow and/or pleura) were found to influence

**Figure 1** Free-from-progression survival of HD patients according to risk groups defined by the initial plasma levels of TNF, p55 and p75. The low-risk group denotes that all three plasma markers were within their normal values ($n$ = 10), while the high-risk group denotes that the plasma levels of TNF, p55 and p75 were above these limits ($n$ = 14). Patients with one or two plasma markers elevated combined to form the intermediate-risk group ($n$ = 37)

**Figure 2** Overall survival of HD patients according to risk groups defined by the initial plasma levels of TNF, p55 and p75. The low-risk group denotes that all three plasma markers were within their normal values ($n$ = 10), while the high-risk group denotes that the plasma levels of TNF, p55 and p75 were above these limits ($n$ = 14). Patients with one or two plasma markers elevated combined to form the intermediate-risk group ($n$ = 37)

© Cancer Research Campaign 1998

**Figure 3** Free-from-progression survival (left-panels) and overall survival (right panels) of HD patients according to the Straus (**A** and **B**), Hasenclever (**C** and **D**), Proctor (**E** and **F**), Manchester (**G** and **H**), Wagstaff (**I** and **J**) and TNF ligand receptor-based (**K** and **L**) indices. In each panel, the box denotes the number of patients in each category defined by the index as published, and the *P* value denotes the result of the log-rank test

*© Cancer Research Campaign 1998*

both FFP and overall survival (log-rank test $P < 0.05$). However, the low number of events of disease progression and death did not allow meaningful multivariate regression analysis for prognostic variables in the present cohort of patients.

### TNF ligand receptor-based prognostic factor model

As a predictive model that summarizes the relationship between the elevated plasma levels of TNF, p55, p75 and outcome of HD, we analysed them in combination rather than as individual plasma markers because of the usefulness of the TNF-based prognostic index in NHL patients (Warzocha et al, 1997). Three risk groups were identified depending on the initial plasma levels of TNF, p55 and p75. The low-risk group denotes that all three plasma markers were within their normal values ($n = 10$), while the high-risk group denotes that the plasma levels of TNF, p55 and p75 were above these limits ($n = 14$). Patients with one or two elevated plasma markers combined to form the intermediate-risk group ($n = 37$). The frequency of a complete response to treatment in the low-, intermediate- and high-risk groups were, respectively, 100%, 92% and 71% ($P < 0.05$). The actuarial 2-year FFP survival rates in these groups were, respectively, 90%, 85% and 56% (log-rank test $P < 0.05$) (Figure 1). Overall survival rates at 2 years in the low-, intermediate- and high-risk groups were 100%, 93%, and 71% respectively (log-rank test $P < 0.01$) (Figure 2).

Using our data set, we evaluated the importance of the TNF ligand receptor-based prognostic index along with five other reported prognostic indices for HD (Wagstaff et al, 1988; Straus et al, 1990; Proctor et al, 1991; Hasenclever et al, 1995; Lee et al, 1997). The TNF ligand receptor-based prognostic index was the only index to have a significant prognostic impact for FFP survival (Figure 3K). Using the same indices for analysis of overall survival, only the simplified Manchester index (low- and high-risk groups) (Lee et al, 1997) and the index derived from the current TNF, p55 and p75 plasma levels analysis were found to have significant predictive values (Log-rank test $P < 0.005$ and $P < 0.01$, respectively) (Figure 3H and L).

### DISCUSSION

In this study, plasma levels of TNF, p55 and p75 were found to be higher in HD patients than in healthy subjects, clearly suggesting their increased production in the course of the disease. H-RS cells have been reported to produce several cytokines and receptors, including those of the TNF ligand receptor family members (Nadali et al, 1994; Gruss and Dower, 1995). Therefore, the association between TNF and its soluble receptors' plasma levels and disease extent and burden suggests the possibility that their plasma levels in HD patients reflect the neoplastic component of the disease. This suggestion is supported by the observations of others who found elevated levels of p55 TNF receptor and soluble CD30 in HD patients (Gruss et al, 1993; Nadali et al, 1994). In addition, the TNF-related ligands may interact with surrounding reactive cells, particularly T cells, which in turn may enhance the production of cytokines and receptors in a paracrine fashion (Gruss and Dower, 1995; Bazzoni and Beutler, 1996). The correlation between TNF plasma levels and the number of monocytes suggests that circulating reactive cells may also contribute to enhanced TNF production in HD patients. Altogether, these data indicate that both malignant and reactive cell populations may ultimately influence TNF and its soluble receptor plasma levels in HD patients. It is noteworthy that patients with the nodular sclerosis histological subtype were found to have significantly lower levels of TNF and, to a lesser extent, of p55 and p75 compared with those with mixed cellularity histology. This observation indicates that a possible heterogeneity of cytokine production in HD may be linked to the histological features of the tumour.

The close associations between the elevation of TNF and its soluble receptors in plasma and the presence of B symptoms, hypergammaglobulinaemia, increased serum levels of fibrinogen and alkaline phosphatase, anaemia and low serum albumin suggest that the TNF ligand receptor system may be functionally involved in the pathophysiological syndrome of inflammation and cachexia associated with HD. Several studies have revealed possible ways in which excessive TNF production could contribute to this phenomenon (Tracey et al, 1988; Lindemann et al, 1989; Denz et al, 1993; Gruss and Dower, 1995). Importantly, all these adverse conditions may result in a poor performance status of the host and may influence the patient's ability to tolerate therapy and, as a consequence, result in a worse prognosis. Upon the stimulation of TNF receptors, several transduction pathways are activated, including the transcription factor nuclear factor kappa B (NF-κB), which leads to the coordinated expression of many proteins involved in further amplification of the inflammatory response. Therefore, TNF is believed to play a pivotal role in persistent immune activation (Bazzoni and Beutler, 1996; Barnes and Karin, 1997). Altogether, the plasma levels of circulating TNF and its receptors seem to reflect several ongoing biological events involved in the neoplastic process, including those related to the tumour, the host and the tumour–host relationship.

Finally, our study indicates that the elevation of TNF and its receptors constitutes an adverse prognostic parameter for HD patients' outcome. Moreover, the combination of these parameters in the prognostic index, already validated for NHL patients, was found to be useful in identifying three categories of HD patients with different outcome. Interestingly, this index identified a subgroup of patients, representing 16% of the entire population, in which no death occurred, although no such subgroup was identified in our population by any other published indices. This population should therefore be considered for therapeutic attitudes that do not expose HD patients to long-term therapy-related complications. On the other hand, the TNF ligand receptor-based index allowed the identification of a quarter of the entire population of patients with a high risk of progression or death, which occurred in about half of the patients of this subgroup. The size of this high-risk group, which is substantially larger than the high-risk groups identified in our population by the Proctor or Manchester indices, allows the targeting of a category of patients in which more aggressive therapeutic attitudes may be worth testing in the course of the disease. Thus, compared with other indices already present for the selection of high-risk patients, the TNF ligand receptor index seems promising both in terms of the size of the subgroups with a particular prognosis and in terms of its statistical significance. It should be mentioned, however, that some of these indices (Straus, Wagstaff and Manchester) were developed for the identification of high-risk patients in advanced HD patients, although the population analysed here was unselected. In addition, because of the limited number of HD patients studied here and of the relatively short follow-up, these results should be considered as being preliminary; a larger prospective study is clearly needed to confirm these encouraging results.

Further studies are also warranted as prognostic indices consist of clinical and biological features that are mostly surrogate variables

© Cancer Research Campaign 1998

for the biological heterogeneity of the disease (Shipp, 1994). This study and those presented by others (Gruss et al, 1993; Nadali et al, 1994; Seymour et al, 1997) clearly indicate the possible use of certain biological markers along with established clinical and laboratory variables for more appropriate prognostic risk assignment in HD patients.

### ACKNOWLEDGEMENTS

This work was supported by the Hospices Civils de Lyon – PHRC (96.044) and INSERM (Paris, ERCA). KW was supported by a grant founded by the Fondation de France (Paris).

### REFERENCES

Barnes PJ and Karin M (1997) Nuclear factor-κB – a pivotal transcription factor in chronic inflammatory diseases. *N Eng J Med* **336**: 1066–1071

Bazzoni F and Beutler B (1996) The tumour necrosis factor ligand and receptor families. *N Eng J Med* **334**: 1717–1725

Denz H, Orth B, Weiss G, Gallati H, Herrmann R, Huber P, Wachter H and Fuchs D (1993) Serum soluble tumor necrosis factor receptor 55 is increased in patients with hematological neoplasias and is associated with immune activation and weight loss. *Eur J Cancer* **29A**: 2232–2235

Gruss HJ and Dower SK (1995) Tumor necrosis factor ligand superfamily: involvement in the pathology of malignant lymphomas. *Blood* **85**: 3378–3404

Gruss HJ, Dolken G, Brach MA, Mertelsmann R and Herrman F (1993) The significance of serum levels of soluble 60 kD receptors for tumor necrosis factor in patients with Hodgkin's disease. *Leukemia* **7**: 1339–1343

Gruss HJ, Ulrich D, Braddy S, Armitage RJ and Dower SK (1995) Recombinant CD30 ligand and CD40 ligand share common biological activities on Hodgkin and Reed–Sternberg cells. *Eur J Immunol* **25**: 2083–2089

Hasenclever D, Schimtz N and Diehl V (1995) Is there a rationale for high dose chemotherapy as first line treatment of advanced Hodgkin's disease? *Leuk Lymph* **15** (suppl. 1): 47–49

Le Hir M, Bluethmann H, Kosco-Vilbois MH, Muller M, di Padova F, Moore M, Ryffel B and Eugster HP (1996) Differentiation of follicular dendritic cells and full antibody responses require tumor necrosis factor receptor-1 signaling. *J Exp Med* **183**: 2367–2372

Lee SM, Radford JA, Ryder WDJ, Collins CD, Deakin DP and Crowther D (1997) Prognostic factors for disease progression in advanced Hodgkin's disease: an analysis of patients aged under 60 years showing no progression in the first 6 months after starting primary chemotherapy. *Br J Cancer* **75**: 110–115

Lindemann A, Ludwig WD, Oster W, Mertelsmann R and Herrmann F (1989) High-level secretion of tumour necrosis factor-alpha contributes to hematopoietic failure in hairy cell leukemia. *Blood* **73**: 880–884

Nadali G, Vinante F, Ambrosetti A, Todeschini G, Veneri D, Zanotti R, Meneghini V, Ricceti MM, Benedetti F, Vassanelli A, Perona G, Chilosi M, Menestrina F, Fiacchini M, Stein H and Pizzolo G (1994) Serum levels of soluble CD30 are elevated in the majority of untreated patients with Hodgkin's disease and correlate with clinical features and prognosis. *J Clin Oncol* **12**: 793–797

Pasparakis M, Alexopoulou L, Episkopou V and Kollias G (1996) Immune and inflammatory responses in TNFα-deficient mice: a critical requirement for TNFα in the formation of primary B cell follicles, follicular dendritic networks and germinal centers, and in the maturation of the humoral immune response. *J Exp Med* **184**: 1397–1411

Proctor SJ, Taylor P, Donnan P, Boys R, Lennard A and Prescott RJ (1991) A numerical prognostic index for clinical use in identification of poor-risk patients with Hodgkin's disease at diagnosis. *Eur J Cancer* **27**: 624–629

Salles G, Bienvenu J, Bastion Y, Barbier Y, Doche C, Warzocha K, Gutowski MC, Rieux C and Coiffier B (1996) Elevated circulating levels of TNFα and its p55 soluble receptor are associated with an adverse prognosis in lymphoma patients. *Br J Haematol* **93**: 352–359

Seymour JF, Talpaz M, Hagemeister FB, Cabanillas F and Kurzrock R (1997) Clinical correlates of elevated serum levels of interleukin-6 in patients with untreated Hodgkin's disease. *Am J Med* **102**: 21–28

Shipp MA (1994) Prognostic factors in aggressive non-Hodgkin's lymphoma: who has 'high risk' disease? *Blood* **83**: 1165–1173

Straus DJ, Gaynor JJ, Myers J, Merke DP, Caravelli J, Chapman D, Yahalom J and Clarkson BD (1990) Prognostic factors among 185 adults with newly diagnosed advanced Hodgkin's disease treated with alternating potentially noncross-resistant chemotherapy and intermediate-dose radiation therapy. *J Clin Oncol* **8**: 1173–1186

Tracey KJ, Wei H, Manogue KR, Fong Y, Hesse DG, Nguyen HT, Kuo GC, Beutler B, Cotran RS and Cerami A (1988) Cachectin/tumor necrosis factor induces cachexia, anemia, and inflammation. *J Exp Med* **167**: 1211–1227

Wagstaff J, Gregory WM, Swindell R, Crowther D and Lister TA (1988) Prognostic factors for survival in stage IIIB and IV Hodgkin's disease: a multivariate analysis comparing two specialist treatment centres. *Br J Cancer* **58**: 487–492

Warzocha K, Bienvenu J, Coiffier B and Salles G (1995) Mechanisms of action of the tumor necrosis factor and lymphotoxin ligand-receptor system. *Eur Cytokine Network* **6**: 83–96

Warzocha K, Salles G, Bienvenu J, Bastion Y, Dumontet C, Renard N, Neidhardt EM and Coiffier B (1997) The tumor necrosis factor ligand-receptor system can predict treatment outcome in lymphoma patients. *J Clin Oncol* **15**: 499–508

*© Cancer Research Campaign 1998*


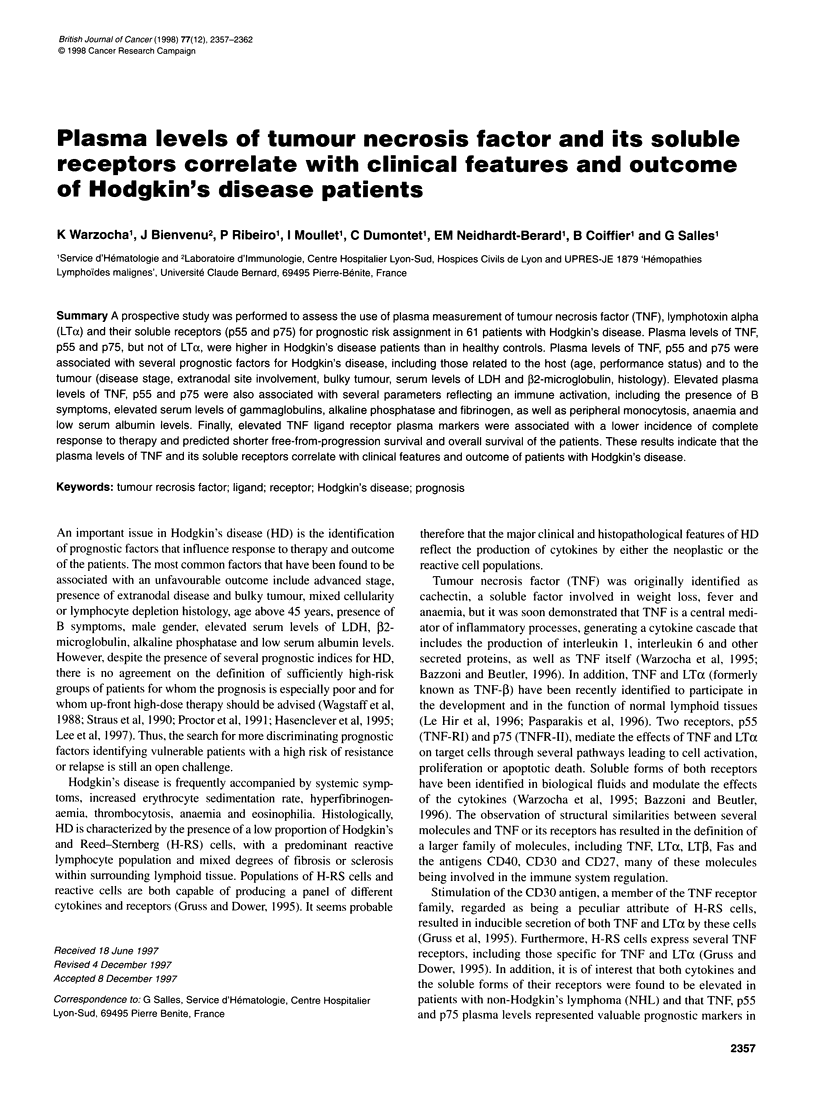

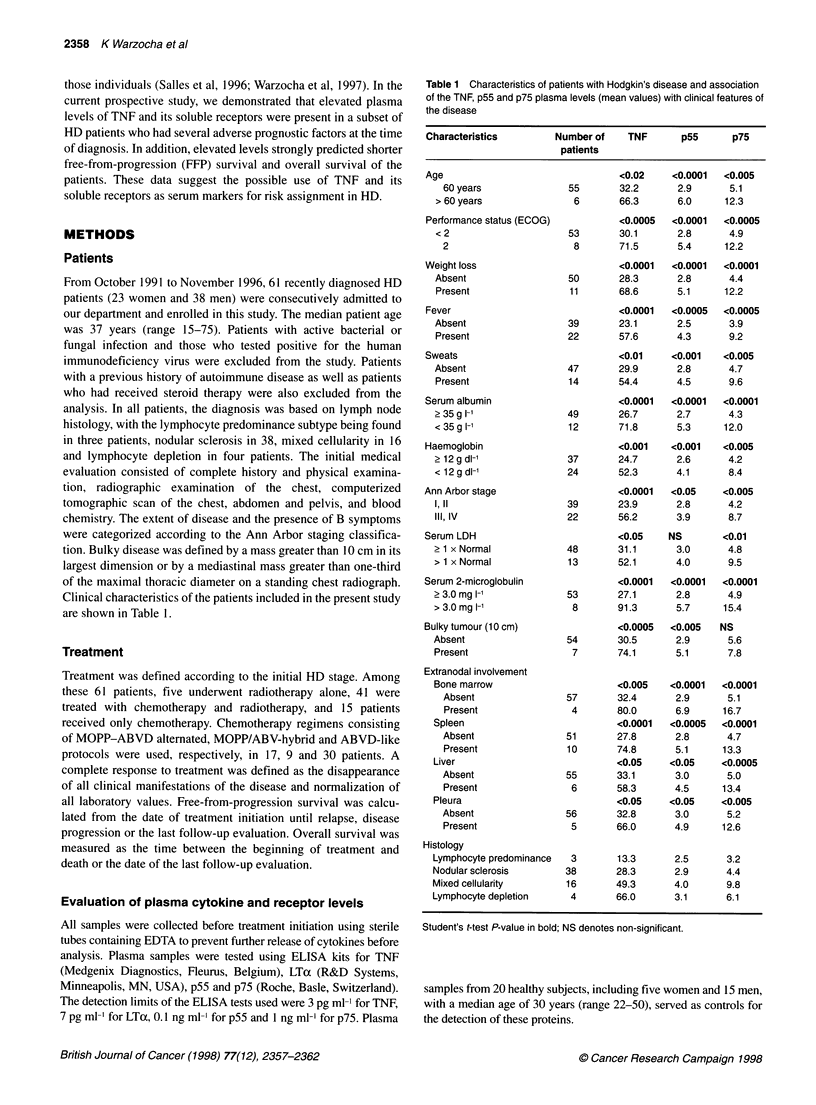

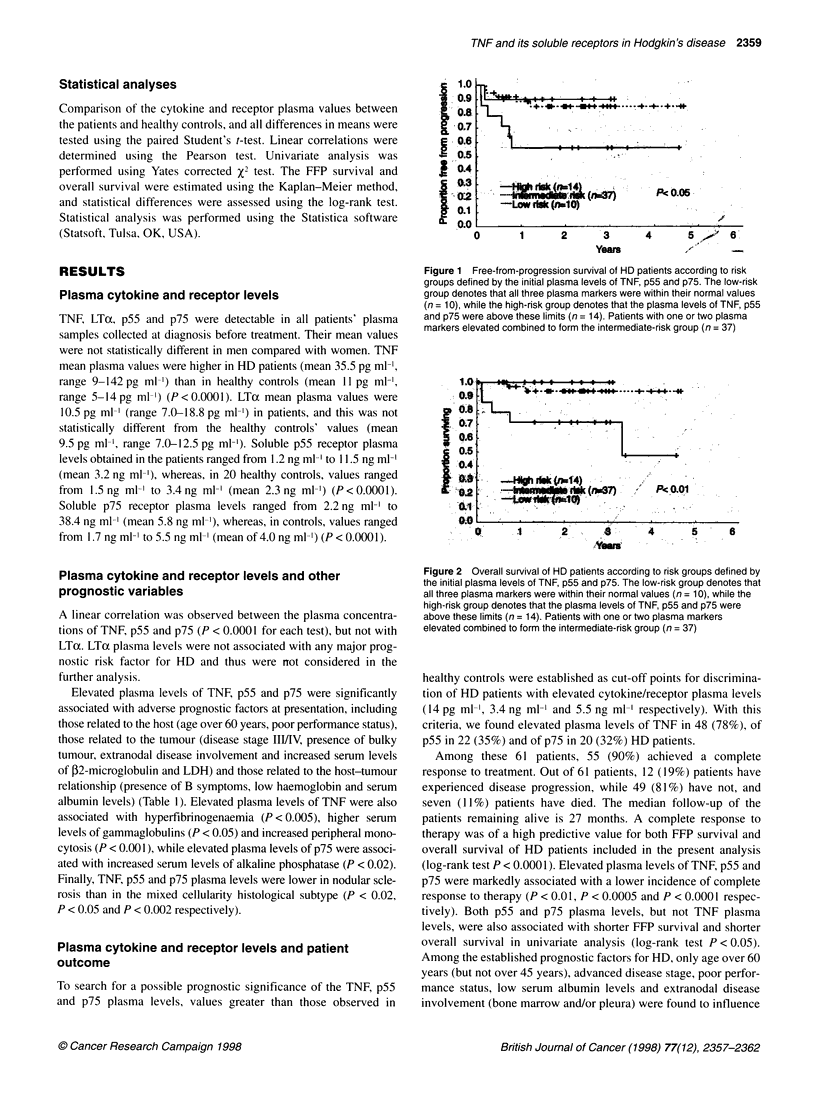

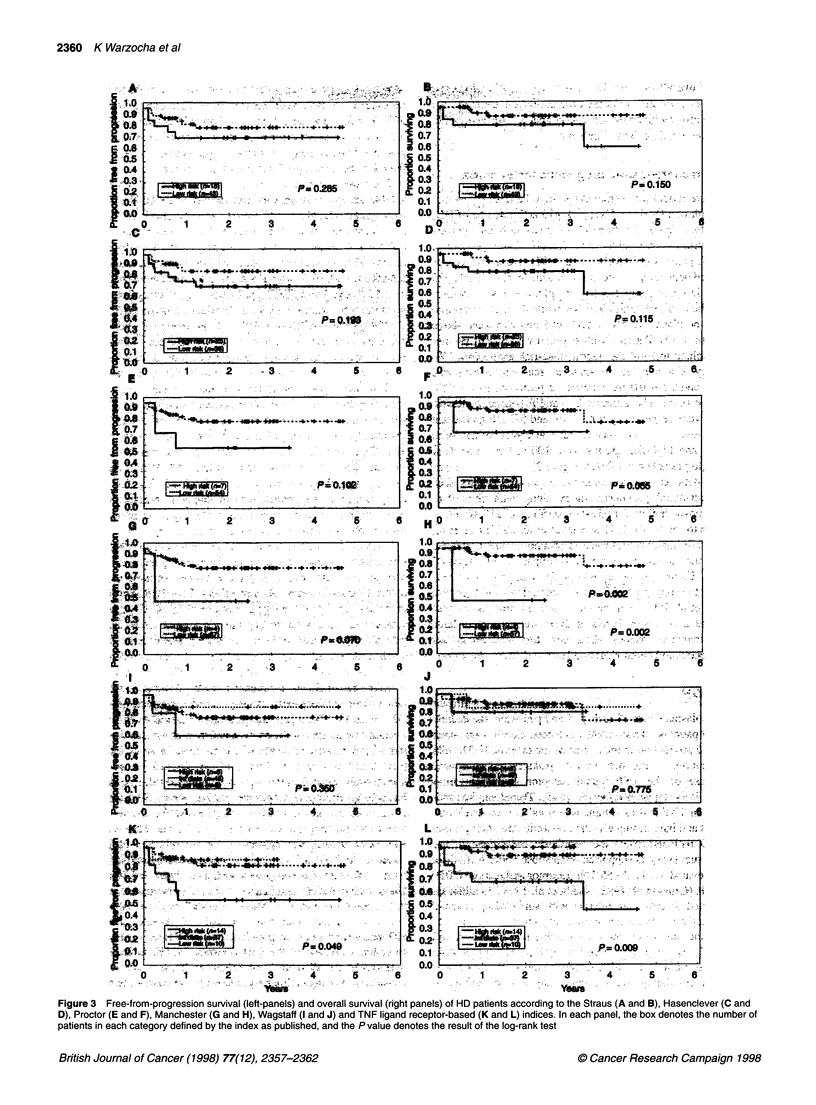

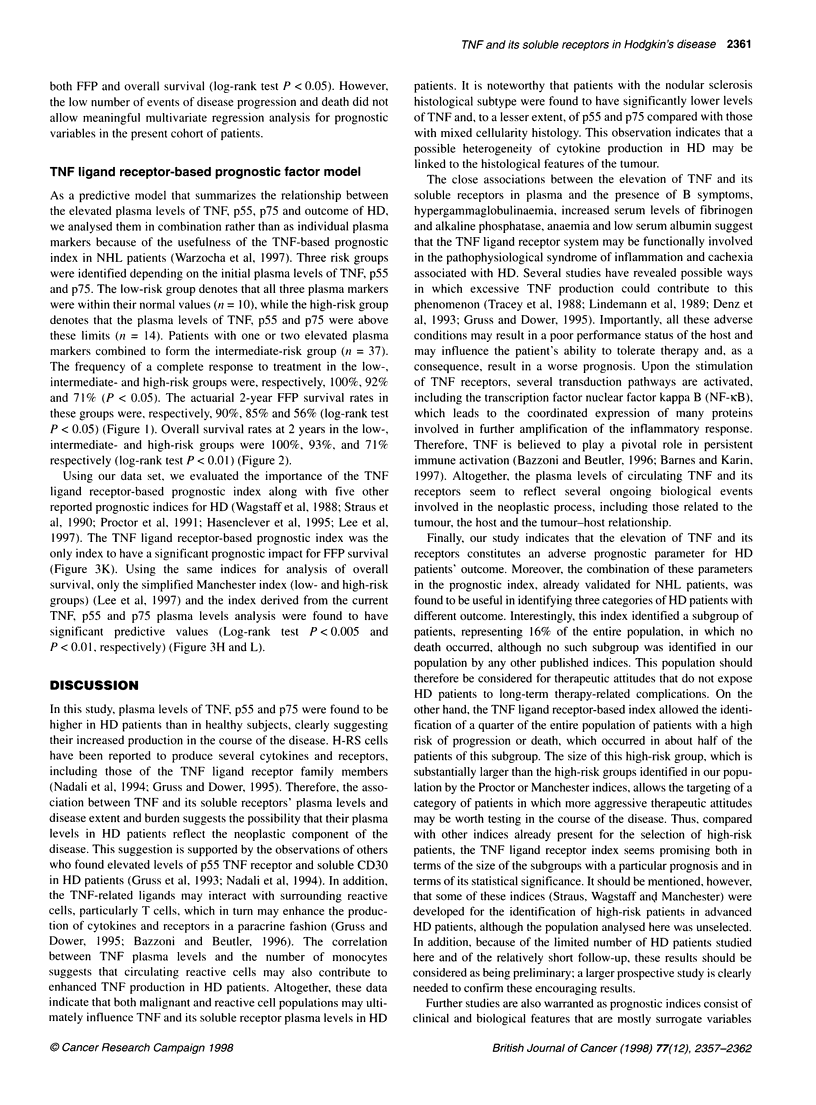

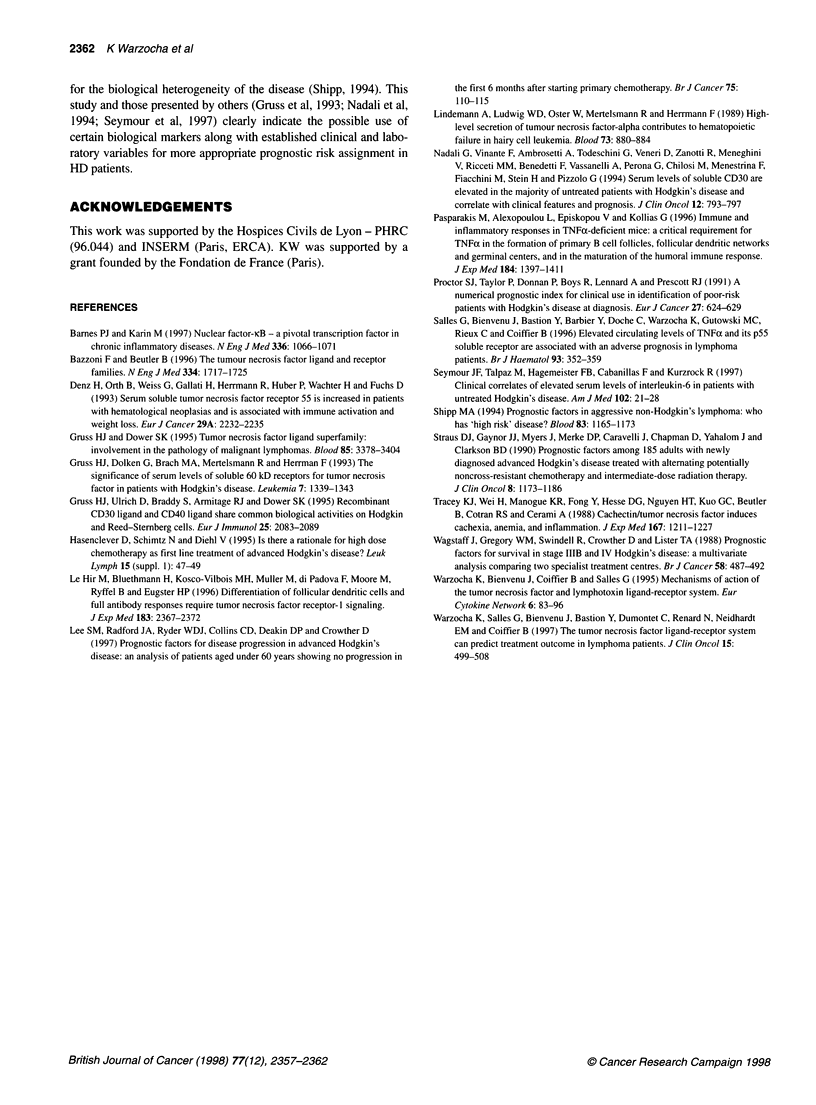


## References

[OCR_00985] Barnes P. J., Karin M. (1997). Nuclear factor-kappaB: a pivotal transcription factor in chronic inflammatory diseases.. N Engl J Med.

[OCR_00989] Bazzoni F., Beutler B. (1996). The tumor necrosis factor ligand and receptor families.. N Engl J Med.

[OCR_00993] Denz H., Orth B., Weiss G., Gallati H., Herrmann R., Huber P., Wachter H., Fuchs D. (1993). Serum soluble tumour necrosis factor receptor 55 is increased in patients with haematological neoplasias and is associated with immune activation and weight loss.. Eur J Cancer.

[OCR_00999] Gruss H. J., Dower S. K. (1995). Tumor necrosis factor ligand superfamily: involvement in the pathology of malignant lymphomas.. Blood.

[OCR_01002] Gruss H. J., Dölken G., Brach M. A., Mertelsmann R., Herrmann F. (1993). The significance of serum levels of soluble 60kDa receptors for tumor necrosis factor in patients with Hodgkin's disease.. Leukemia.

[OCR_01007] Gruss H. J., Ulrich D., Braddy S., Armitage R. J., Dower S. K. (1995). Recombinant CD30 ligand and CD40 ligand share common biological activities on Hodgkin and Reed-Sternberg cells.. Eur J Immunol.

[OCR_01012] Hasenclever D., Schmitz N., Diehl V. (1995). Is there a rationale for high-dose chemotherapy as first line treatment of advanced Hodgkin's disease? German Hodgkin's Lymphoma Study Group (GHSG).. Leuk Lymphoma.

[OCR_01017] Le Hir M., Bluethmann H., Kosco-Vilbois M. H., Müller M., di Padova F., Moore M., Ryffel B., Eugster H. P. (1996). Differentiation of follicular dendritic cells and full antibody responses require tumor necrosis factor receptor-1 signaling.. J Exp Med.

[OCR_01023] Lee S. M., Radford J. A., Ryder W. D., Collins C. D., Deakin D. P., Crowther D. (1997). Prognostic factors for disease progression in advanced Hodgkin's disease: an analysis of patients aged under 60 years showing no progression in the first 6 months after starting primary chemotherapy.. Br J Cancer.

[OCR_01032] Lindemann A., Ludwig W. D., Oster W., Mertelsmann R., Herrmann F. (1989). High-level secretion of tumor necrosis factor-alpha contributes to hematopoietic failure in hairy cell leukemia.. Blood.

[OCR_01037] Nadali G., Vinante F., Ambrosetti A., Todeschini G., Veneri D., Zanotti R., Meneghini V., Ricetti M. M., Benedetti F., Vassanelli A. (1994). Serum levels of soluble CD30 are elevated in the majority of untreated patients with Hodgkin's disease and correlate with clinical features and prognosis.. J Clin Oncol.

[OCR_01044] Pasparakis M., Alexopoulou L., Episkopou V., Kollias G. (1996). Immune and inflammatory responses in TNF alpha-deficient mice: a critical requirement for TNF alpha in the formation of primary B cell follicles, follicular dendritic cell networks and germinal centers, and in the maturation of the humoral immune response.. J Exp Med.

[OCR_01052] Proctor S. J., Taylor P., Donnan P., Boys R., Lennard A., Prescott R. J. (1991). A numerical prognostic index for clinical use in identification of poor-risk patients with Hodgkin's disease at diagnosis. Scotland and Newcastle Lymphoma Group (SNLG) Therapy Working Party.. Eur J Cancer.

[OCR_01057] Salles G., Bienvenu J., Bastion Y., Barbier Y., Doche C., Warzocha K., Gutowski M. C., Rieux C., Coiffier B. (1996). Elevated circulating levels of TNFalpha and its p55 soluble receptor are associated with an adverse prognosis in lymphoma patients.. Br J Haematol.

[OCR_01063] Seymour J. F., Talpaz M., Hagemeister F. B., Cabanillas F., Kurzrock R. (1997). Clinical correlates of elevated serum levels of interleukin 6 in patients with untreated Hodgkin's disease.. Am J Med.

[OCR_01068] Shipp M. A. (1994). Prognostic factors in aggressive non-Hodgkin's lymphoma: who has "high-risk" disease?. Blood.

[OCR_01072] Straus D. J., Gaynor J. J., Myers J., Merke D. P., Caravelli J., Chapman D., Yahalom J., Clarkson B. D. (1990). Prognostic factors among 185 adults with newly diagnosed advanced Hodgkin's disease treated with alternating potentially noncross-resistant chemotherapy and intermediate-dose radiation therapy.. J Clin Oncol.

[OCR_01080] Tracey K. J., Wei H., Manogue K. R., Fong Y., Hesse D. G., Nguyen H. T., Kuo G. C., Beutler B., Cotran R. S., Cerami A. (1988). Cachectin/tumor necrosis factor induces cachexia, anemia, and inflammation.. J Exp Med.

[OCR_01085] Wagstaff J., Gregory W. M., Swindell R., Crowther D., Lister T. A. (1988). Prognostic factors for survival in stage IIIB and IV Hodgkin's disease: a multivariate analysis comparing two specialist treatment centres.. Br J Cancer.

[OCR_01090] Warzocha K., Bienvenu J., Coiffier B., Salles G. (1995). Mechanisms of action of the tumor necrosis factor and lymphotoxin ligand-receptor system.. Eur Cytokine Netw.

[OCR_01095] Warzocha K., Salles G., Bienvenu J., Bastion Y., Dumontet C., Renard N., Neidhardt-Berard E. M., Coiffier B. (1997). Tumor necrosis factor ligand-receptor system can predict treatment outcome in lymphoma patients.. J Clin Oncol.

